# TiO_2_ Nanotube Arrays: Fabricated by Soft–Hard Template and the Grain Size Dependence of Field Emission Performance

**DOI:** 10.1186/s11671-017-2361-9

**Published:** 2017-11-13

**Authors:** Xuxin Yang, Pei Ma, Hui Qi, Jingxin Zhao, Qiang Wu, Jichun You, Yongjin Li

**Affiliations:** 1 Hangzhou Normal University, No. 16, Xuelin Str. Xiasha High-education Zone, Hangzhou, 310036 People’s Republic of China; 2 The Second Hospital of Jilin University, Changchun, 130041 People’s Republic of China; 3 Key Laboratory of Mesoscopic Chemistry of MOE, School of Chemistry and Chemical Engineering, Nanjing University, Nanjing, 210093 People’s Republic of China

**Keywords:** Nanocrystalline materials, Polymers, Surfaces, Microstructure

## Abstract

Highly ordered TiO_2_ nanotube (TNT) arrays were successfully synthesized by the combination of soft and hard templates. In the fabrication of them, anodic aluminum oxide membranes act as the hard template while the self-assembly of polystyrene-block-poly(ethylene oxide) (PS-b-PEO) complexed with titanium-tetraisopropoxide (TTIP, the precursor of TiO_2_) provides the soft template to control the grain size of TiO_2_ nanotubes. Our results indicate that the field emission (FE) performance depends crucially on the grain size of the calcinated TiO_2_ which is dominated by the PS-b-PEO and its blending ratio with TTIP. The optimized sample (with the TTIP/PEO ratio of 3.87) exhibits excellent FE performances involving both a low turn-on field of 3.3 V/um and a high current density of 7.6 mA/cm^2^ at 12.7 V/μm. The enhanced FE properties can be attributed to the low effective work function (1.2 eV) resulted from the smaller grain size of TiO_2_.

## Background

One-dimensional nanomaterials have attracted great interest due to their potential for numerous applications, e.g., electron field emitter [1–5]. TiO_2_ nanotubes (TNTs) are promising candidate for the emitter due to the high aspect ratio, low work function (4.5 eV), and high oxidation resistance [4]. The nanotube diameters, height, wall thickness, and density as well as the regularity of the nanoarray dependences of the field emission (FE) performance have been investigated in detail [6, 7]. A significant number of nanotube arrays are available by the aid of the development of the synthetic approaches [8, 9]. Especially, the template strategies have been widely employed to fabricate nanotube array. For instance, Tsai et al. prepared diamond nanotip arrays with various sizes and periods by anodic aluminum oxide (AAO) [10]. During the preparation, the micro-channels in AAO membrane can act as an excellent hard template to induce the formation of highly ordered nanoarrays. In the synthesis of porous TiO_2_ nanofibers in our previous work, the self-assembly of block copolymer has been proved as an effective template for the selective distribution and the grain size manipulation of TiO_2_ [11]. The highly ordered TNT arrays with tunable grain sizes can be expected by the combination of the soft and hard templates. For one thing, it is facile to tailor the diameter, center-to-center distance, and the length of the TiO_2_ arrays by means of various AAO membranes; for another thing, the wall thickness, grain size, and the density of the TiO_2_ nanotubes are under the control of the block copolymer and the precursor of TiO_2_. Most importantly, the structure control in TNT array and tube levels can be performed separately. In this work, therefore, the TiO_2_ arrays with various grain sizes have been fabricated in the blend of titanium-tetraisopropoxide (TTIP)/block copolymer. In addition to the hard template (AAO) for the formation of highly ordered arrays, the PS-b-PEO is employed as the soft template to control the grain size of TiO_2_. The field emission performances of the resultant TNT arrays exhibit obvious grain size dependence, which has been attributed to the variation of the effective work function.

## Methods

**Scheme 1:**
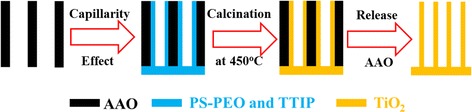
Preparation of TNT arrays with the combination of soft and hard templates

The porous AAO membrane (Whatman, Germany) with the pore size of ~ 200 nm and the thickness of 60 μm and polystyrene-block-poly(ethylene oxide) (Sigma-Aldrich, USA) with a molecular weight of 58,500–37,000, 58,600–71,000, and 60000–14,500 g/mol were used. Titanium-tetraisopropoxide (TTIP, Sigma-Aldrich, USA) acts as the precursor of TiO_2_. PS-b-PEO and TTIP were dissolved in chloroform with various composition ratios (Table 1). S1 to S5 are samples corresponding to the indicated block copolymer and the blend ratio. For instance, S1 was prepared using the block copolymer of M_w_ = 58,500–37,000 and the TTIP/PEO blend ratio of 3.87. After stirring for 5 h at room temperature, the mixed solution was transferred to the bottom of AAO membranes. The solution can go into the channels in AAO upon the capillary effect. Then, the samples were dried at 120 °C for 12 h in vacuum. After calcined at 450 °C for 2 h in air, the samples were immersed in NaOH solution (3 mol/L) for 1 h to remove the alumina frame. Finally, the products were washed with deionized water and dried at 40 °C for 24 h (Scheme 1).

**Table 1:** Samples with various molecular weights of PS-b-PEO and its blending ratio with TTIP

Sample name	S1	S2	S3	S4	S5
PS-PEO	58,500–37,000	58,500–37,000	58,500–37,000	60,000–14,500	58,600–71,000
TTIP/PEO^a^	3.87	5.16	10.32	3.64	3.70
Grain size (nm)^b^	10.7	12.5	14.9	11.8	13.5

^a^TTIP/PEO represents the weight ratio between TTIP and PEO in PS-b-PEO

^b^Grain sizes were calculated from XRD profiles shown in Fig. 2

A Hitachi S-4800 FESEM was used for morphology measurement at an accelerating voltage of 5.0 kV. The X-ray diffraction (smartlab3, Rigaku Japan) data were collected at a scanning speed of 2°/min with a step interval of 0.02°. The electron field emission measurements were carried out using a diode configuration, a cathode (sample), and a parallel anode plate at a distance of 150 μm in a vacuum chamber (2 × 10^−6^ Torr).

## Results and Discussion

**Fig. 1:**
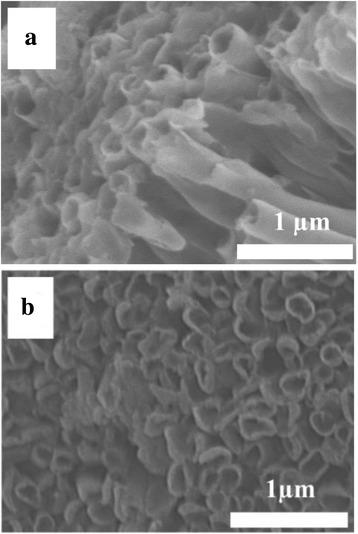
SEM images of obtained TNTs from the side (**a**) and *top* (**b**) view

**Fig. 2:**
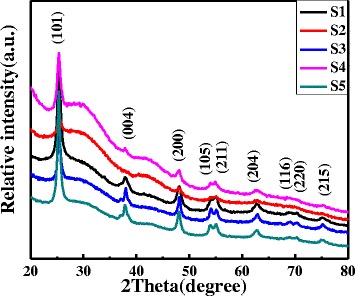
XRD profiles of TNT arrays

Figure 1 shows the typical SEM images of TNT arrays by taking S1 as an example (all samples exhibit similar morphologies). In the SEM image of the side view (Fig. 1a), there are some vertically aligned nanotubes with the diameter of ~ 200 nm. Figure 1b illustrates the SEM images of the top view of TNT arrays, in which the diameter of the nanotubes can be further confirmed. Figure 2 shows the XRD profiles of all samples dried at 40 °C for 24 h. There are strong diffraction peaks locating at 25°, 38°, and 48°, which agrees well with the reported values of anatase TiO_2_ from JCPDS Card No. 84-1286. All samples exhibit strong preferential growth orientations along the (101) plane (25°). The mean grain sizes were calculated from the full width at half maximum (FWHM) of the (101) diffraction peaks using the Debye–Scherrer formula [12]:

$$ D=0.9\lambda /{\beta}_{2\theta}\cos \theta $$

where *D*, *λ*, *β*_2*θ*_, and *θ* are the mean grain size, X-ray wavelength (1.5418 Å), FWHM in radians, and Bragg’s diffraction angle, respectively. The sample grain sizes are listed in Table 1. Obviously, the increase of TTIP weight fraction in the blend (from S1 to S3) results in the higher magnitude of grain size.

**Fig. 3:**
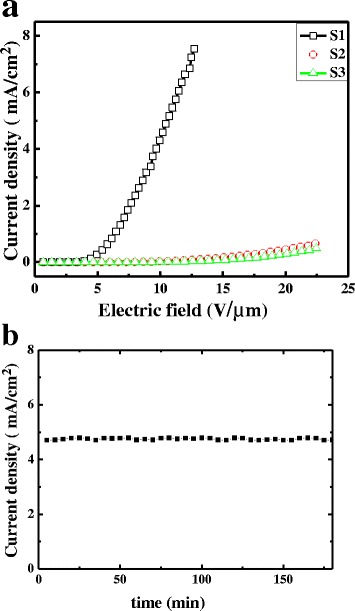
Current density–electric field (*J–E*) plot (**a**) and the current density stability of S1 under 10 V μm^−1^ for 180 min (**b**)

**Fig. 4:**
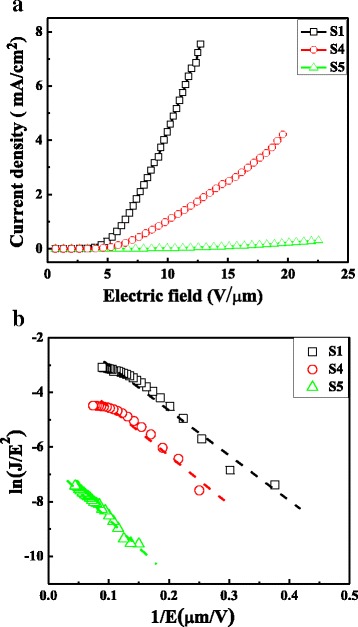
**a** Current density–electric field (*J–E*) plot. **b** The corresponding Fowler–Nordheim plots

Fowler–Nordheim (F–N) theory is usually used to further analyze the FE properties of the TNT arrays [13]. It can be expressed as *J* = (*Aβ* ^2^ *E* ^2^/*φ*) exp(−*Bφ* ^3/2^/*βE*), where *J* is the FE current density (A/cm^2^), *E* is the applied electric field (V/μm), *∅* is the work function (4.5 eV for TiO_2_), *β* is the field enhancement factor related to the emitter geometry, and *A* and *B* are constants whose values are 1.56 × 10^−6^(A eV V^−2^) and 6.83 × 10^3^(eV^−3/2^ V μm^−1^), respectively. Figure 3a illustrates the current density–electric field (*J–E*) plot of TNT cathodes, which exhibit exponential dependence. The turn-on field and the threshold field are defined as the electric field at an emission current density of 0.01 and 1.0 mA/cm^2^, respectively. For S1, the turn-on field and threshold field are 3.3 and 6.4 V/μm, respectively, with excellent cycle stability as shown in Fig. 3b. However, the turn-on fields are 10.3 and 13.2 V/μm for S2 and S3, respectively. No threshold voltage is observed in the results of S2 and S3 within the studied electric field range. To clarify the reason for the great difference of field emission performance among them, our attention is paid to the different nanotube thicknesses and grain sizes of TiO_2_ obtained in XRD profiles. For one thing, the thicknesses (estimated in SEM images, data not shown here) are 24, 29, and 36 nm in S1, S2, and S3, respectively. For another thing, the grain sizes of anatase TiO_2_ obtained from XRD profiles are 10.7 (S1), 12.5 (S2), and 14.9 nm (S3) as shown in Table 1. To distinguish the roles of tube thickness and grain size in the field emission performance, the nanotubes with the similar thickness were prepared based on the blend ratios shown in Table 1. Figure 4a represents the field emission characteristic of these samples under an applied bias voltage. The average turn-on fields (obtained from at least three samples) of S1, S4, and S5 are 3.3 ± 0.4, 4.2 ± 0.3, and 8.7 ± 0.5 V/μm, respectively. Although there are kinds of parameters influencing field emission performance, it is still reasonable to attribute the different field emission performances to the grain size since the samples exhibiting the similar tube thickness were fabricated according to the same condition. Furthermore, the smaller size (10.7 nm for S1) corresponds to the lower turn-on field (3.3 V/μm). It is worth to notice that S1 exhibits the maximum current density as large as 7.6 mA/cm^2^ at the field of 12.7 V/μm which is much higher than the reported values while the turn-on field is comparable with the results in references [14–18].

The FE behavior of TNTs can be modeled following the well-known Fowler–Nordheim (FN) equation, as shown in Fig. 4b. The good linear fit in the curves indicates that the field emission current originates only from barrier tunneling electrons extracted by the electric field. Based on the slope of the FN plot (*k*)_,_ it is facile to calculate the effective work functions using the following equation:

*k* =  − (6.83 × 10^3^)*φ* ^3/2^/*β*.

They are 1.2, 1.5, and 2.1 eV for S1, S4, and S5, respectively, by assuming the field enhancement factor (pristine TNT arrays) is 445 [18]. The reduction in the turn-on electric field of the TNTs is caused by the decrease of the effective potential barrier height resulted from the smaller TiO_2_ grains. Therefore, it is reasonable to attribute the enhanced field performance to the grain boundary effect and resultant up-shift of Fermi level which can be interpreted as follows [4, 19]. Polycrystalline materials are composed of small nanocrystalline grains separated by grain boundaries, which lead to a large number of grain boundary defects. These defects are benefit for both electron trapping and electron supply due to the effective conducting pathway. This is the reason for the increase of carrier concentration and subsequent up-shift of Fermi level [19]. This rising Fermi level can reduce the work function (Fig. 4b) and the effective potential barrier height of TNTs, corresponding to easy electron emission, accounting for the enhanced field emission performance.

## Conclusions

The TNT arrays were synthesized by the combination of soft and hard templates. On one hand, the AAO membranes induce the vertically aligned nanotubes. On the other hand, both the block copolymer and its blend ratio with TTIP produce remarkable influence on the grain size of the TiO_2_. The relationship between the grain size and the FE performance has been clarified for the first time. Our results indicate that the decrease of grain size accounts for the stronger grain boundary conduction, leading to the lift of the Fermi level. This is the reason for the lower work function, the smaller effective potential barrier, and the resultant-enhanced FE performance.
